# Abusive behavior of employees against their managers: an explorative study

**DOI:** 10.3389/fpsyg.2025.1576385

**Published:** 2025-05-02

**Authors:** Galit Avisar, Tami Cohen, Aharon Tziner, Hadara Bar-Mor

**Affiliations:** ^1^Netanya Academic College, Netanya, Israel; ^2^Netanya Academic College, Tel-Hai College and Peres Academic Center, Tel Hai, Israel

**Keywords:** upward abusive behavior, abusive behavior, misbehavior, abusive behavior by gender, abusive behavior by age

## Abstract

**Objective:**

Workplace abusive behavior is behavior that causes harm to people’s dignity and their mental and physical health. It encompasses abusive or violent behaviors, harassment, or intimidation, and can be directed against co-workers, managers, customers, or suppliers. Extant literature has focused mainly on abuse by managers, or by peers and colleagues. This study investigates ‘upward abusive behavior’“– abuse by an employee against people in management positions, and whether there is awareness of or exposure to this phenomenon. While not common, abusive behavior from employees toward their supervisors can manifest through disrespectful conduct and inappropriate challenges to authority, creating a difficult work environment.

**Method:**

120 employees and managers from a variety of organizations, and with varying seniority, answered a questionnaire (based on Tepper, 2000) that included 15 statements relating to the conduct and dynamics between employees and managers. The statements were assessed on a Likert scale of 1 (“I cannot remember him/her ever using this behavior with me”) to 5 (“He/she uses this behavior very often with me”).

**Results:**

Several significant associations were found between managers’ exposure to abuse and some of the respondents’ demographic data such as age, gender, seniority in the workplace, and tenure.

**Conclusion:**

The result of this exploratory study indicates that many employees and managers are aware of the existence and various aspects of upward abuse as it is expressed in organizations. However, they still do not give it an explicit name or are not willing to acknowledge it. The phenomenon of employee abuse of managers is not only not recognized in legislation (as is the phenomenon of regular workplace abusive behavior), but it is also not discussed in organizations. There are no procedures or processes to prevent and eradicate it.

## 1 Introduction

The purpose of the current study was to examine the abusive behavior of employees against managers in the workplace. The general term ‘workplace abusive behavior ‘or ‘workplace bullying’ encompasses abusive or violent behavior, harassment, or intimidation. The identification and awareness of its incidence have been increasing in recent years, according to a ruling of the National Labor Court (Yithak Khakmon vs. the State of Israel, 21,934-02-21, September 6, 2022). It can be directed against subordinates, co-workers, managers, customers, or suppliers. Workplace abuse has been named ‘the silent epidemic’ because many of the victims prefer not to complain, and employers avoid dealing with it (Meiri, 2013).

This is unacceptable behavior, and damages not only the people who experience it but also the work environment and the entire organization. Abusive behavior manifests on repeated occasions and may occur at different levels in the organization and between different types of employees. This phenomenon has been studied extensively (e.g., Tziner et al., 2024). Still, the focus has mostly been on two types of abusive behavior – abuse of employees by managers, and peer bullying by colleagues. Studies have shown that workplace abuse harms employees’ satisfaction with the workplace, their functioning and productivity, and their mental and physical health (Branch et al., 2007). However, employee abuse of managers – ‘upward abusive behavior’ – has so far not been thoroughly examined, and few studies can be found in the literature. However, informal evidence reveals that the phenomenon of reverse abusive behavior does exist. This aim of this exploratory study was to empirically examine the prevalence and scope of upward abusive behavior and the degree to which employees and managers in various organizations are aware of it. We also attempted to examine whether the phenomenon of reverse workplace abusive behavior has similar parameters to the abuse of employees by managers and whether it is affected by various conditions such as type of organization, seniority of the employees involved, and more.

### 1.1 Workplace abusive behavior

The phenomenon of workplace abusive behavior began to receive academic attention in the 1980s, mainly due to studies that pointed to the impact of repetitive abusive behaviors on employees and organizations. In the 1990s, research on workplace bullying gained momentum in the academic and social world. Study spread from the Scandinavian countries to other countries, mainly in Western Europe and the United States. The studies found that employees who experienced workplace abusive behavior experienced higher rates of stress, anxiety, depression, and other health problems. After the 1990s, workplace abusive behavior became a subject that was studied more deeply in terms of health effects, organizational influences, and prevention methods. Since the 2000s, researchers’ interest has continued to develop. Today, there is a broader understanding of this phenomenon, which includes the health and social effects of workplace abusive behavior as well as its impact on the organization. The latest studies deal not only with the behavior itself but also with the effects and implications of organizational culture and the nature of employment on the existence of the phenomenon and the implications for the physical and mental health of employees. In recent years, we have been aware of changes in the world of employment such as technological and economic changes, which form a change in the conduct of organizations and the nature of employment. For example, remote work during and after the COVID-19 pandemic has led to changes and new ways to identify and prevent workplace bullying (Tziner et al., 2022).

#### 1.1.1 What is workplace abusive behavior?

There is no universally agreed definition of workplace abusive behavior, and there are many different definitions in the literature. However, it can be said that workplace abusive behavior is psychological abuse that involves humiliation and attempts to sabotage someone’s work. From the managerial aspect, there may be excessive surveillance by the manager or unjustified criticism or abuse by colleagues such as defamation of a co-worker to other employees or superiors, ostracism of co-workers, or humiliation in front of others. These are negative behaviors – direct and indirect – of aggression, hostility, intimidation, and harm that are ongoing and involve individuals or groups, whether privately or publicly, whether face-to-face or virtually. While the COVID-19 period has brought new challenges and fewer face-to-face interactions, namely working from home behind screens, even during this period the abuse has moved to the electronic media, especially to the various WhatsApp groups of employees. Although this interaction is not face-to-face, the ‘light hand on the keyboard’ allows employees to bully and be bullied in these cases as well. Employees or managers are exposed to these negative behaviors, which are repeated and intended to harm them, by harassing or insulting them and causing feelings of humiliation and stress, alienation, or social ostracism. Experts (e.g., Huh et al., 2025) define workplace abusive behavior as a series of undesirable behaviors that may harm the individual psychologically or physically. To be defined as workplace abusive behavior, they must occur frequently, repeatedly, and for a specified period of time. Examples of behaviors that have an abusive dimension include public humiliation, spreading rumors, social alienation, outbursts of anger, and even threats. Beyond the personal dimension, abusive behavior touches on aspects of the work itself such as questioning the employee’s professionalism, blocking vital information, and more. A conflict or argument between an employee and a manager, or between two co-workers, does not turn the situation into abuse. Some studies (in Goldenberg-Aharoni et al., 2019) define abuse as having four parts or elements that coexist and turn the behavior into abuse. The first three elements include inappropriate and negative behavior, which occurs over time and frequently, and the negative behavior increases – both in its negative impact and in its intensity. The fourth element, which we expand on later, speaks of asymmetry in the relationship between the two sides. The tendency to think that when both sides have an equal balance of power there is no abuse, is an approach that has characterized studies in the past. This perception limits the observation and understanding of the phenomenon of workplace abusive behavior as a whole. Another look at workplace bullying notes three main types of hostile actions in the workplace according to their level. The first defines behavior that is expressed in impoliteness – the offenders direct their insults toward certain individuals and address them in unprofessional terms, anger, and contempt. Such behavior can lead to dissatisfaction with the job to the point of wanting or intending to quit the workplace. The second behavior is harassment, which includes immoral and systematic actions, which repeatedly cause the victims to feel helpless. This behavior manifests in various forms such as defamation, excessive monitoring of work performance, and unreasonable criticism. This is behavior that is often based on race, religion, gender, age, or a disability, which can harm the physical and mental health of the object of the abuse. The third type is characterized by negative verbal and non-verbal behavior that are repeated over a long period of time. This type of abuse is characterized by deliberate and long-term abuse – which includes harm and insult, social exclusion, emotional abuse, humiliation in private or public, deliberate ignorance, and even gossip and spreading rumors about him. Here, too, the abuse is based on personal characteristics such as race, religion, gender, age, or disability. The existing theoretical frameworks for defining bullying are based on one of the following two concepts: harm to human dignity, or harm to mental and physical health and safety. But these frameworks are not enough to capture the injustice of bullying, and offer little guidance to policymakers in designing anti-bullying regulation. Schneebaum (2021) proposes another legal theory, based on Weber (1978) conception of officers’ authority and its definition as an abuse of organizational power. This approach opens up new possibilities for shaping the prohibitions of bullying. At the same time, this approach draws our attention to the deep challenges involved in regulating bullying, in ways that have been ignored until now.

Various studies (Ferris et al., 2008; Fischer et al., 2021; Camps et al., 2020; Choi, 2019; Eissa and Lester, 2017) have noted the damage caused to victims of workplace bullying, to both their physical and mental health, and the extensive impact it has on the functioning of employees in the workplace. Employees who experience abuse show decreased motivation and productivity. These workers lose their commitment to work, which has a wide impact on the workplace. Beyond the effect of the immediate decrease in productivity, an employee who experiences abuse feels alienated and unwilling to work together in a team, and this leads to a murky atmosphere in the workplace that also affects other employees. The impact of workplace abusive behavior spills over from the workplace and affects other areas of the employee’s life, whether in the family or social framework. Another consequence of workplace abusive behavior is peer abuse by colleagues, who learn and imitate undesirable behaviors of managers toward employees and perform such behaviors themselves. The entire team is exposed to the manager’s undesirable attitude and behavior toward a team member, and this permeates and affects the behavior of other team members. Over the years, the phenomenon of workplace abusive behavior has expanded, and the understanding and research of the issue has increased around the world (Bai et al., 2022). Workplace abusive behavior exists in organizations, not only in the context of a manager toward an employee, but also between employees and their colleagues and, as mentioned, upward abusive behavior from employee to manager, which we discuss below.

#### 1.1.2 The importance of defining and identifying workplace abuse

The crucial importance of work in an individual’s and society’s life requires creating defense mechanisms for all those involved in the employment sphere. One of the key challenges is finding the delicate balance between the employers’ right to run their business as they see fit, and the employees’ rights and needs.

The work environment, despite its importance, can become fertile ground for negative behaviors that allow certain individuals to abuse others. The abuse can be vertical –managers to their employees, or horizontal – between coworkers. Therefore, a double defense system is required. First, mechanisms that *a priori* prevent misconduct and, second, treatment and penalty tools in cases of abuse. The effectiveness of these mechanisms relies largely on the willingness of employees to disclose abuse incidents. However, although the phenomenon of workplace abuse has become common in many places of work, employees often avoid filing complaints. The reasons lie in their fear that their complaints would not get the proper attention, and that the very act could lead to additional abuse or damage to their employment status and future.

Labor courts have recommended using the occurrence of workplace abuse as a mechanism of injury at work to receive compensation from the National Insurance Institute in the context of social security (see the case of Rina Shabtai vs. the National Security Institute, 13,230-05-23, September 24, 2024).

## 2 Various aspects of workplace abuse

### 2.1 Managers’ abuse of employees

Abuse by managers includes a wide range of abusive behaviors by managers toward their employees. This abuse can be expressed in various behaviors: public or private humiliation, abusive employment, and inappropriate bullying (see the case X vs. Y1, Y2 and the Israeli Police, 41,516-01-22, November 11, 2023), the use of offensive language, disrespect for the employee’s skills, and insulting him or her in front of others. Workplace abusive behavior directly related to work can be expressed by preventing promotion or recognition, ignoring the employee’s achievements, and preventing his promotion despite meeting goals, setting impossible demands, removing important information, creating an unreasonable workload, or setting unrealistic goals. Such abuse can also go as far as professional isolation or social isolation within the group, threats, and intimidation with various punishments, and even the threat of dismissal.

Possible reasons for managers’ abusive behavior include several factors. a. Personality factors: Managers who bully employees are often characterized by dominance, control, and lack of empathy. These traits, combined with the need to increase their sense of power and status in the organization, lead to dominant and aggressive behavior. These managers often justify their behavior as a way to ensure that goals are met or as a response to personal pressures. b. Pressures and an organizational environment: Work environments characterized by heightened pressure, unrealistic goals, and a lack of resources can encourage abusive behavior. Managers may experience pressure to deliver results quickly, leading to the use of force in an unethical manner. c. Poor organizational culture: An organizational culture that has no clear mechanisms to prevent abusive behavior or to punish the abuser. In these cases, workplace abusive behavior can become the norm, especially in organizations where managers perceive their behavior as unquestionable. In addition, organizations that do not encourage open communication or provide emotional and occupational support to employees increase the possibility of workplace abusive behavior (Bai et al., 2022).

Over the years, many studies (see in Bhattacharjee and Sarkar, 2024) have been conducted on the subject, and some have proposed solutions for reducing the phenomenon. Those include training employees and management how to identify, prevent, and support cases of abuse; improving organizational policies and strictly enforcing workplace behavior rules and open communication regarding problem-solving procedures; providing psychological support for victims of abuse including hotlines and counseling; conducting periodic surveys regarding employee satisfaction and identifying problems of workplace abusive behavior; implementing an anti-abusive behavior strategy by defining specifically prohibited behaviors; providing employees and managers with tools to identify abusive behaviors and respond to them with an emphasis on consistent enforcement and defined sanctions for those involved in abusive behavior (Park, 2023; Woodrow and Guest, 2017).

The issue of hierarchical relationships in the workplace and the power inherent in the authority of the office are issues that have not been sufficiently regulated. It is claimed that abusive behavior creates a sense of job insecurity when the abuser is a manager or supervisor and affects the behavior of the rest of the team members toward the victimized employee. Employees who witness their direct manager’s behavior may feel anger toward the manager’s behavior is unjustified, or are satisfied or agree with this behavior because they feel that the victim deserves it. Beyond this passive reaction, a manager’s abusive behavior can also have an active impact on the team members. It can often serve as a role model if that manager is appreciated and admired. Employees tend to imitate positive or negative behavior by a manager who is a role model. Abusive behavior can lead to social and organizational learning, and could eventually result in peer abuse. Such conduct could become an organizational culture, in which managers behave badly to their employees, and team managers or middle managers learn from it and, in turn, behave this way toward their employees. This behavior could be interpreted as acceptable and legitimate in the organization. The result of passive or active exposure can result in reduced performance, unwillingness to be creative, decreased productivity, and less commitment to the organization.

### 2.2 Abuse by colleagues or peers

As mentioned, abusive behavior has implications beyond how it affects the victim. Third party employees can imitate the bullying of a member of the team, or the victim himself can conclude that this sort of behavior is acceptable and legitimate. Abusing co-workers is a phenomenon about which there is relatively little research compared to abusive behavior by a manager. Although about 75% of reported abuse relates to workplace abusive behavior by a manager, in recent years the research has been expanded to examine what happens between colleagues within the organization and its effect on the individual and the organization (Tziner et al., 2024). Most workplace interactions exist between employees, so it is likely that cases of abuse occur in these interactions. Abuse by colleagues is hostile behavior by employees toward colleagues who are at the same level of responsibility or authority in the organization. It can include a variety of harmful behaviors that damage the victim emotionally, psychologically, and sometimes physically. Abuse by colleagues can occur in the form of gossip, spreading rumors, negative talk behind one’s back, and disparaging criticism. It also includes social exclusion – deliberately ignoring the victims or excluding them from social events and group activities at work, which leads to loneliness and a sense of alienation. Another form is psychological abuse – constant humiliation, doubling the work of a particular employee, and denying access to vital information or resources. Peer abuse can be sustained by threats and constant emotional pressure. Coworkers can behave in a covert abusive manner by ignoring requests for help by a team member, refusing to collaborate on projects, or holding employees accountable for mistakes at work, in a way that puts the employee in an uncomfortable position. Abusive behavior between colleagues can cause victims significant mental harm, such as depression, anxiety, decreased self-confidence, and impaired productivity. Sometimes, employees who are abused choose to leave the workplace (Lutgen-Sandvik et al., 2010).

When a co-worker commits the abuse, a sense of occupational vulnerability and job insecurity may be created. Also, according to the theory of social interaction, abuse by co-workers affects solidarity and morale in the workplace. In most cases, there are psychological consequences, often invisible, but usually have traumatic consequences. This behavior violates the norms of behavior in organizations, leads to decreased performance and creates a toxic work environment. Employees with a proactive personality might take the initiative and act independently to influence the environment, face challenges such as abusive behavior and try to improve the situation, instead of waiting for things to happen on their own. This behavior can reduce the negative effects of abusive management by managers or workplace abusive behavior by colleagues. A person with a less proactive personality may feel helpless to change the hostile relationship with managers and colleagues.

Workplace satisfaction is an issue of great importance today, and it has been proven that there is a direct connection between it and effectiveness and productivity at work (Cheyroux et al., 2024). On the other hand, dissatisfaction can lead to a lack of motivation and a lack of commitment to the workplace. Workplace abusive behavior by colleagues can reduce the victim’s satisfaction and thus affect his or her conduct.

### 2.3 Upward abusive behavior – abuse of managers by employees

There is scant literature and research on the subject of upward abusive behavior –abuse by an employee against his or her manager in the workplace. Despite the increase in research on workplace abusive behavior in recent decades, the main focus has been on ‘downward workplace abusive behavior’ (by managers) with some recent attention paid to ‘horizontal workplace abusive behavior’ (by peers). The phenomenon of upwards abusive behavior, in which managers experience abusive behavior by a members of their team has so far not received sufficient research attention; for example, the case of X vs. Y (20284-07-23, May 6, 2024), in which the plaintiff filed a claim under the Defamation Prohibition Law-1965, based on a number of the manager’s statements. The complaint included “… insubordinate, does whatever he wants, allows himself to bully the manager, and is unfit for an organizational framework.” The claim was rejected, but legitimized the manager’s criticism of the employee as part of “his duty and right to comment on employees’ work and criticize their performance as an integral part of proper manpower management, and as a common interest of both the employees and the employer.”

Some documentation claims that some of the potential factors that contribute to upwards workplace abusive behavior include the work environment, changes within the organization, and power issues. Despite the widespread opinion that workplace abusive behavior is usually done by managers toward employees, there is an understanding that managers can indeed be the target of workplace abusive behavior by their employees (Fischer et al., 2021; Camps et al., 2020). Organizational status can affect the possibility of abusive behavior in the workplace from the perspective of team members. The power that derives from organizational status can stem from mastery of information and knowledge or professional expertise. A manager who faces an ‘expert’ is at a disadvantage and could become the target of social, negative, and systematic actions. Thus, the multidirectionality of power and the changing sources of power within organizations raise additional questions; specifically, whether power relates directly to one’s formal authority. The team relies on managers for resources and rewards, while managers depend on the team to be productive and accomplish the organization’s goals. However, the balance is upset when one side denies or interferes with the other in achieving its goals. For example, team members can acquire power when they withhold information from the manager, which prevents them from achieving their managerial goals. Moreover, the strength of a team member is especially significant when it is difficult to replace their knowledge, skills, and expertise. The manager’s dependence on the team provides the team with power that they can abuse. These behaviors fit the definition of workplace abusive behavior if they are persistent or constitute an ongoing threat. Consequently, just as the manager can abuse employees, the team or an employee can abuse the manager’s dependence when it comes to the fulfillment of his goals and the goals of the organization. It seems, therefore, that factors such as organizational change and dependence on employees may play a role in the occurrence of upward workplace abusive behavior. The work environment and organizational culture are fertile grounds for the creating this phenomenon. Factors such as work pressure, an inefficient work environment, lack of cooperation between team members, normalization of inappropriate behaviors, and a shortage of workforce are some of the factors that contribute to an inadequate work environment. A manager whose position is not respected may be more exposed to workplace abusive behavior. Moreover, a lack of support from senior management may also exacerbate the situation. Understandably, workplace abusive behavior can occur at all levels of the organization, and managers are also exposed to abusive behavior by an employee or employees – whether disguised or overt.

### 2.4 Ways to deal with workplace abusive behavior

Ways to deal with the phenomenon of workplace abusive behavior usually include clear policies and procedures in organizations, which include anonymous reporting mechanisms, training and workshops for managers and employees, personal and emotional support for employees, and more. The expansion of the phenomenon and its recognition emphasizes the need to promote a culture of respect in the workplace, to create support and cooperation, to prevent harmful behaviors, and to create a positive and productive work environment. Organizations must dedicate resources to preventing and dealing with this phenomenon to support the well-being of employees and the success of the organization.

Regulation of workplace abusive behavior exists in various countries around the world, but it differs from country to country. There are countries in the world where there is specific legislation to deal with this phenomenon, and in others, the phenomenon is dealt with through existing legislation for another issue – criminal or tort around harassment or workplace abusive behavior, with different emphases in the regulations. In Israeli law, there is no explicit law prohibiting workplace abusive behavior, except the Prevention of Workplace Abusive Behavior Bill 5,782-2022 (which has not yet come into effect) defines workplace abusive behavior as repetitive behavior toward an individual, on several separate occasions, which can create a hostile work environment, including one or more of these behaviors. The bill also defines the various forms of behavior that can be constituted as bullying: a demeaning, humiliating or harmful attitude; damage to employment conditions because of unwarranted reasons; undermining one’s ability to do his job, including by setting unreasonable demand or creating unreasonable conditions to perform it; subordinating someone to an atmosphere of fear and threats including by shouting, false accusations or spreading harmful rumors; accrediting others with the victim’s work and achievements or blaming him/her for others’ failures; and professional or social isolation at work.

Although the bill has not been ratified, labor courts recognized the injustice of workplace abusive behavior and awarded compensation for mental anguish or breach of the duty to act in good faith. The prohibition on workplace abusive behavior is based on the Basic Law: Human Dignity and Liberty, the increased duty of good faith in labor law, and the employer’s obligation to provide employees with a fair and safe work environment.

## 3 Method

In the current survey, we distributed a questionnaire based on the Tepper questionnaire (2020). We used Google Forms to formulate the questionnaire, with an introduction explaining the nature of the questionnaire and its purpose. The questionnaire was distributed in a WhatsApp group and a group of master’s degree students, as well as groups of HR managers and on Facebook to our associates. The questionnaire was answered by 120 respondents of both genders, from a variety of organizations, and with varying seniority. After closing the response, we collected the findings and began the work of analyzing the data.

### 3.1 Participants

The study population included 77 women (64.2%) and 43 men (35.8%), aged 22–76 (mean 44.09, SD 10.64). Most participants were employed in private organizations (55.8%), public organizations (25.0%), and government organizations (14.2%), and had 0.5–42 years of experience. Most of the participants are academics (70.8%) and most had tenure at work (63.9%). Nearly half of the respondents were managers (45.4%). Table 1 below shows the distribution of the participants according to the demographic variables.

**Table 1:** Demographics of participants (*N* = 120).

Demographic	Options	*N*	%	Min.	Max.	Mean	SD
Gender	Male	77	64.2				
	Female	43	35.8				
Age				22	76	44.09	10.64
Education	High school	18	15.0				
	Tertiary	17	14.2				
	BA	46	38.3				
	MA and higher	39	32.5				
Job tenure (years)	Yes	43	36.1				
	No	76	63.9				
Job seniority				0.5	42.0	14.3	10.94
Management position	Yes	65	54.6				
	No	54	45.4				
Type of organization	Private	67	55.8				
	Public	30	25.0				
	Government	17	14.2				
	Other	6	5.0				
Gender of manager	Female	53	44.2				
	Male	67	55.8				

### 3.2 Research tool

The questionnaire was made up of two parts – a demographic questionnaire and the questionnaire based on Tepper (2000). The demographic data included gender, age, education, seniority at work, tenure, managerial position, type of organization (private, public, or governmental), and the gender of the manager. The second part of the questionnaire was based on the Tepper Questionnaire (2000), which included 15 statements on a Likert scale of 1–6 that characterize the respondent’s level of exposure to employee abuse of his/her manager in the workplace; a sample statement: “Employee humiliates the manager in front of others.” A high value in these statements expresses a high level of exposure to abuse. Table 2 depicts the means and SD of the questionnaire statements.

**Table 2:** Means and SD of questionnaire statements (*N* = 120).

Statement	Range	Mean	SD
Employee humiliates manager in front of others.	1–5	1.78	0.98
Employee thinks that manager’s thoughts or feelings are stupid.	1–6	3.41	1.16
Employe deliberately ignores manager.	1–5	2.51	1.15
Employee makes fun of the manager.	1–5	2.11	1.17
Employee invades the manager’s privacy.	1–6	1.84	1.21
Employee reminds the manager of past mistakes and failures.	1–5	2.07	1.02
Employee does not appreciate the manager’s good performance of tasks that require effort.	1–6	2.83	1.45
Employee attributes his/her own mistakes to the manager.	1–6	2.57	1.33
Employee breaks promises s/he made to the manager.	1–6	2.70	1.33
Employee vents his/her anger on the manager for reasons not concerned with the manager.	1–6	2.50	1.33
Employee makes negative remarks about the manager to others.	1–6	3.38	1.32
Employee is rude to the manager.	1–6	2.12	1.16
Employee does not enable the manager’s proper interactions with coworkers.	1–6	1.83	0.96
Employee tells the manager that s/he is incompetent.	1–6	1.36	0.71
Employee lies to the manager.	1–6	2.93	1.29

In addition, we calculated the general characteristics of the answers to the 15 statements. Table 3 presents the reliability of the exposure to abusive behavior measure.

**Table 3:** Reliability of exposure to abusive behavior measure (*N* = 120).

Measure	Number of statements	Range	Mean	SD	α
Exposure to abusive behavior	15	1.07–4.73	2.40	0.79	0.912

The reliability of the statements (Cronbach’s *α*) was high, meaning a high degree of stability and consistency in the responses.

## 4 Results

To examine the relationships between the demographic variables and the index of exposure to abuse, t-tests for independent samples, variance tests (ANOVA), and Pearson tests were performed. The following tables show the results of the tests (Table 4).

**Table 4:** Exposure to abusive behavior by gender.

Statement	Women (*N* = 77)	Men (*N* = 43)	*t*
Employee humiliates manager in front of others.	1.79 (0.102)	1.77 (0.92)	0.13
Employee thinks that manager’s thoughts or feelings are stupid.	3.36 (1.11)	3.49 (1.24)	0.57
Employe deliberately ignores manager.	2.47 (1.19)	2.58 (1.10)	0.52
Employee makes fun of the manager.	2.10 (1.20)	2.12 (1.14)	0.06
Employee invades the manager’s privacy.	1.79 (1.24)	1.93 (1.16)	0.60
Employee reminds the manager of past mistakes and failures.	1.95 (0.97)	2.28 (1.08)	1.72
Employee does not appreciate the manager’s good performance of tasks that require effort.	2.70 (1.42)	3.07 (1.49)	1.34
Employee attributes his/her own mistakes to the manager.	2.47 (1.30)	2.74 (1.36)	1.10
Employee breaks promises s/he made to the manager.	2.57 (1.28)	2.93 (1.39)	1.43
Employee vents his/her anger on the manager for reasons not concerned with the manager.	2.49 (1.39)	2.51 (1.24)	0.07
Employee makes negative remarks about the manager to others.	3.44 (1.32)	3.28 (1.33)	0.64
Employee is rude to the manager.	2.16 (1.16)	2.07 (1.16)	0.39
Employee does not enable the manager’s proper interactions with coworkers.	1.79 (0.98)	1.91 (0.92)	0.63
Employee tells the manager that s/he is incompetent.	1.31 (0.73)	1.44 (0.67)	0.97
Employee lies to the manager.	2.97 (1.33)	2.84 (1.23)	0.56
Exposure to abusive behavior measure	2.36 (0.81)	2.46 (0.76)	0.70

No significant differences were found for exposure to abusive behavior by gender. The responses of men and women to the statements, and in particular the general index, were non-significant.

Next, Pearson correlations between exposure to abuse and age and seniority were performed (Table 5).

**Table 5:** Pearson correlations between exposure to abuse, and age and seniority.

Statement	Age	Seniority
Employee humiliates manager in front of others.	−0.121	−0.045
Employee thinks that manager’s thoughts or feelings are stupid.	−0.050	−0.016
Employe deliberately ignores manager.	−0.155	−0.047
Employee makes fun of the manager.	−0.171	−0.122
Employee invades the manager’s privacy.	−0.098	−0.030
Employee reminds the manager of past mistakes and failures.	−0.048	0.031
Employee does not appreciate the manager’s good performance of tasks that require effort.	−0.104	−0.066
Employee attributes his/her own mistakes to the manager.	−0.114	0.017
Employee breaks promises s/he made to the manager.	−0.203*	−0.101
Employee vents his/her anger on the manager for reasons not concerned with the manager.	−0.120	0.040
Employee makes negative remarks about the manager to others.	−0.057	−0.054
Employee is rude to the manager.	−0.275**	−0.138
Employee does not enable the manager’s proper interactions with coworkers.	−0.154	−0.025
Employee tells the manager that s/he is incompetent.	−0.042	0.028
Employee lies to the manager.	−0.288**	−0.200*
Exposure to abusive behavior measure	−0.201*	−0.076

*p* < 0.01**, *p* < 0.05*.

A significant negative association was found (*r* = −0.203, *p* < 0.05) between the statement “employee breaks promises s/he made to the manager” and age, so that the older the employees, the less they believes that employees break promises they made to the manager. Similarly, it was found that the older the employees are (*r* = −0.288, *p* < 0.01) and the more senior (*r* = −0.200, *p* < 0.05), the less likely that they believe that employees lie to their manager. In general, it was found that there is a significant negative relationship (*r* = −0.201, *p* < 0.05) between the index of exposure to abuse and age, so the older the employee is, the less s/he is to exposed abuse.

Table 6 depicts the results of the association between exposure to abusive behavior and education.

**Table 6:** Exposure to abusive behavior by education.

Statement	Non-academic	BA degree	MA degree	*F*
Employee humiliates manager in front of others.	1.63 (0.91)	1.74 (1.02)	1.97 (0.99)	1.23
Employee thinks that manager’s thoughts or feelings are stupid.	3.29 (1.15)	3.46 (1.15)	3.46 (1.14)	0.27
Employe deliberately ignores manager.	2.46 (1.07)	2.35 (1.18)	2.74 (1.19)	1.30
Employee makes fun of the manager.	2.14 (1.09)	1.85 (1.15)	2.38 (1.23)	2.28
Employee invades the manager’s privacy.	1.74 (1.15)	1.98 (1.50)	1.77 (0.84)	0.48
Employee reminds the manager of past mistakes and failures.	1.77 (0.88)	2.13 (1.07)	2.26 (1.04)	2.29
Employee does not appreciate the manager’s good performance of tasks that require effort.	2.37 (1.57)	3.02 (1.45)	3.03 (1.27)	2.57
Employee attributes his/her own mistakes to the manager.	2.37 (1.31)	2.46 (1.44)	2.87 (1.17)	1.58
Employee breaks promises s/he made to the manager.	2.54 (1.34)	2.63 (1.50)	2.92 (1.09)	0.86
Employee vents his/her anger on the manager for reasons not concerned with the manager.	2.40 (1.14)	2.59 (1.67)	2.49 (1.05)	0.19
Employee makes negative remarks about the manager to others.	3.46 (1.31)	3.24 (1.42)	3.49 (1.23)	0.44
Employee is rude to the manager.	2.06 (1.11)	2.09 (1.33)	2.23 (0.99)	0.25
Employee does not enable the manager’s proper interactions with coworkers.	1.77 (0.91)	1.74 (1.04)	2.00 (0.89)	0.89
Employee tells the manager that s/he is incompetent.	1.37 (0.69)	1.37 (0.88)	1.33 (0.48)	0.04
Employee lies to the manager.	2.91 (1.36)	2.89 (1.39)	2.97 (1.14)	0.04
Exposure to abusive behavior measure	2.29 (0.72)	2.37 (0.91)	2.53 (0.70)	0.91

No significant differences were found for exposure to abusive behavior by education.

Next, we examined the association between exposure to abusive behavior and tenure (Table 7).

**Table 7:** Exposure to abusive behavior by job tenure (*N* = 120).

Statement	No (*N* = 43)	Yes (*N* = 76)	*t*
Employee humiliates manager in front of others.	1.79 (1.06)	1.78 (0.95)	0.08
Employee thinks that manager’s thoughts or feelings are stupid.	3.33 (1.27)	3.46 (1.10)	0.61
Employe deliberately ignores manager.	2.28 (1.16)	2.63 (1.14)	1.61
Employee makes fun of the manager.	2.00 (1.05)	2.14 (1.23)	0.65
Employee invades the manager’s privacy.	1.70 (0.94)	1.92 (1.34)	0.96
Employee reminds the manager of past mistakes and failures.	2.05 (1.00)	2.08 (1.04)	0.17
Employee does not appreciate the manager’s good performance of tasks that require effort.	2.79 (1.44)	2.87 (1.47)	0.28
Employee attributes his/her own mistakes to the manager.	2.42 (1.30)	2.64 (1.35)	0.89
Employee breaks promises s/he made to the manager.	2.67 (1.36)	2.17 (1.32)	0.14
Employee vents his/her anger on the manager for reasons not concerned with the manager.	2.23 (1.27)	2.66 (1.36)	1.68
Employee makes negative remarks about the manager to others.	3.30 (1.30)	3.43 (1.35)	0.52
Employee is rude to the manager.	1.95 (0.97)	2.22 (1.25)	1.22
Employee does not enable the manager’s proper interactions with coworkers.	1.58 (0.76)	1.97 (1.03)	2.18*
Employee tells the manager that s/he is incompetent.	1.33 (0.57)	1.38 (0.78)	0.41
Employee lies to the manager.	2.84 (1.21)	2.97 (1.35)	0.55
Exposure to abusive behavior measure	2.28 (0.71)	2.46 (0.84)	1.15

p < 0.05*..

As can be seen, in reply to the statement “Employee does not enable the manager’s proper interactions with coworkers,” employees with tenure agreed (1.97) more than employees without tenure (1.58) (t (117) = 2.18, *p* < 0.05). No differences were found in the other statements and in the general index.

T-tests were performed to examine exposure to abusive behavior by management position. The results are presented in Table 8.

**Table 8:** Exposure to abusive behavior by management position.

Statement	Manager		
	No (*N* = 65)	Yes (*N* = 54)	t
Employee humiliates manager in front of others.	1.75 (0.98)	1.81 (0.99)	0.34
Employee thinks that manager’s thoughts or feelings are stupid.	3.48 (1.11)	3.33 (1.23)	0.67
Employe deliberately ignores manager.	2.48 (1.16)	2.54 (1.16)	0.28
Employee makes fun of the manager.	2.03 (1.13)	2.17 (1.21)	0.63
Employee invades the manager’s privacy.	1.60 (1.06)	2.13 (1.33)	2.42*
Employee reminds the manager of past mistakes and failures.	1.95 (0.96)	2.20 (1.09)	1.33
Employee does not appreciate the manager’s good performance of tasks that require effort.	2.75 (1.48)	2.94 (1.43)	0.71
Employee attributes his/her own mistakes to the manager.	2.34 (1.22)	2.83 (1.42)	2.05*
Employee breaks promises s/he made to the manager.	2.57 (1.30)	2.85 (1.37)	1.15
Employee vents his/her anger on the manager for reasons not concerned with the manager.	2.28 (1.34)	2.78 (1.30)	2.06*
Employee makes negative remarks about the manager to others.	3.28 (1.38)	3.52 (1.27)	0.99
Employee is rude to the manager.	1.94 (1.03)	2.35 (1.28)	1.96
Employee does not enable the manager’s proper interactions with coworkers.	1.75 (0.90)	1.93 (1.03)	0.97
Employee tells the manager that s/he is incompetent.	1.42 (0.85)	1.30 (0.50)	0.91
Employee lies to the manager.	2.77 (1.21)	3.11 (1.38)	1.44
Exposure to abusive behavior measure	2.29 (0.74)	2.52 (0.84)	1.56

p < 0.05*..

The results of the three statements “Employee invades the manager’s privacy,” “Employee attributes his/her own mistakes to the manager,” and “Employee vents his/her anger on the manager for reasons not concerned with the manager” were positively significant (*p* < 0.05). That is to say, managers, more than junior employees, believe that employees abuse managers from these three aspects.

Next, we examined the association between abusive workplace behavior and type of organization. Table 9 depicts the results.

**Table 9:** Exposure to abusive behavior by type of organization (*N* = 114).

Statement	Private (*N* = 67)	Public (*N* = 30)	Government (*N* = 17)	*F*
Employee humiliates manager in front of others.	1.69 (0.97)	1.87 (1.01)	2.06 (1.09)	1.06
Employee thinks that manager’s thoughts or feelings are stupid.	3.37 (1.23)	3.50 (1.04)	3.29 (1.05)	0.20
Employe deliberately ignores manager.	2.42 (1.16)	2.50 (1.17)	2.94 (1.09)	1.41
Employee makes fun of the manager.	2.06 (1.20)	2.00 (1.11)	2.35 (1.11)	0.54
Employee invades the manager’s privacy.	1.85 (1.22)	1.87 (1.20)	2.00 (1.37)	0.10
Employee reminds the manager of past mistakes and failures.	2.09 (1.07)	1.81 (0.90)	2.24 (1.09)	0.80
Employee does not appreciate the manager’s good performance of tasks that require effort.	2.73 (1.42)	2.90 (1.56)	2.88 (1.41)	0.17
Employee attributes his/her own mistakes to the manager.	2.42 (1.32)	2.60 (1.30)	2.76 (1.39)	0.54
Employee breaks promises s/he made to the manager.	2.75 (1.40)	2.77 (1.30)	2.59 (1.18)	0.11
Employee vents his/her anger on the manager for reasons not concerned with the manager.	2.48 (1.41)	2.57 (1.36)	2.59 (1.18)	0.07
Employee makes negative remarks about the manager to others.	3.48 (1.32)	3.20 (1.42)	3.18 (1.07)	0.66
Employee is rude to the manager.	2.15 (1.16)	2.23 (1.25)	2.06 (1.14)	0.12
Employee does not enable the manager’s proper interactions with coworkers.	1.75 (0.89)	2.03 (1.10)	1.94 (1.03)	0.99
Employee tells the manager that s/he is incompetent.	1.39 (0.82)	1.23 (0.43)	1.59 (0.71)	1.34
Employee lies to the manager.	2.93 (1.37)	2.90 (1.06)	2.94 (1.52)	0.01
Exposure to abusive behavior measure	2.37 (0.80)	2.40 (0.85)	2.49 (0.78)	0.16

No significant differences were found between the responses of the respondents from different types of organizations, in any of the statements, and in particular in the general index.

Next, we examined the relationship between exposure to abusive behavior in the workplace and the manager’s gender. Results are shown in Table 10.

**Table 10:** Exposure to abusive behavior by manager’s gender.

Statement	Women (*N* = 53)	Men (*N* = 67)	*t*
Employee humiliates manager in front of others.	1.96 (1.09)	1.65 (0.90)	1.66
Employee thinks that manager’s thoughts or feelings are stupid.	3.27 (1.22)	3.49 (1.09)	1.01
Employe deliberately ignores manager.	2.55 (1.22)	2.49 (1.11)	0.26
Employee makes fun of the manager.	2.14 (1.17)	2.05 (1.17)	0.41
Employee invades the manager’s privacy.	2.14 (1.52)	1.67 (0.88)	1.96
Employee reminds the manager of past mistakes and failures.	1.98 (0.99)	2.11 (1.06)	0.67
Employee does not appreciate the manager’s good performance of tasks that require effort.	2.88 (1.44)	2.73 (1.46)	0.56
Employee attributes his/her own mistakes to the manager.	2.73 (1.52)	2.35 (1.11)	1.52
Employee breaks promises s/he made to the manager.	2.82 (1.41)	2.65 (1.27)	0.69
Employee vents his/her anger on the manager for reasons not concerned with the manager.	2.71 (1.53)	2.37 (1.18)	1.34
Employee makes negative remarks about the manager to others.	3.41 (1.42)	3.32 (1.23)	0.38
Employee is rude to the manager.	2.27 (1.31)	2.06 (1.05)	0.96
Employee does not enable the manager’s proper interactions with coworkers.	1.90 (1.08)	1.81 (0.88)	0.50
Employee tells the manager that s/he is incompetent.	1.35 (0.82)	1.40 (0.64)	0.32
Employee lies to the manager.	3.20 (1.39)	2.70 (1.21)	2.04
Exposure to abusive behavior measure	2.49 (0.91)	2.32 (0.71)	1.09

As it turns out, female managers are more likely than male managers to believe that employees lies to their managers.

## 5 Discussion

This exploratory study examined the existence of the phenomenon of upward abusive behavior – employee abuse of managers. As mentioned, this is a phenomenon that has not been thoroughly studied to date. Most of the research (Bai et al., 2022; Escartín, 2016; Fischer et al., 2021; Lev-Wiesel et al., 2023; Lutgen-Sandvik et al., 2010; Woodrow and Guest, 2017) has focused on the abuse of employees by managers, and recently there have been studies dealing with the relationships between colleagues at work and the abuse that occurs in the relationships (peer abuse). Examining and researching this topic is important and should be studied in depth, to obtain a broad picture of the interpersonal relationships in the organization. Just as the relationship and abuse committed by managers toward their employees has been investigated, it is appropriate to investigate the opposite phenomenon – the abuse of managers by employees. Interactions between managers and employees happen all the time in organizations and have a decisive impact on the satisfaction of both employees and managers, productivity levels, mental health, and more (Liang et al., 2022; Schaufeli et al., 2008). Managers’ satisfaction in organizations is key to organizational success. Managers are those who move the organization forward, manage the day-to-day of their employees and are responsible for meeting the organization’s goals as defined by top management. Therefore, it is particularly important to examine the issue of workplace abusive behavior by employees toward managers.

This study sought to examine whether there is awareness or exposure to the existence of the employee abuse of managers.

First, the Abuse Exposure Index (Tziner et al., 2022) was examined – an average for each subject for all the responses to the 15 statements: an average of 2.40 with a standard deviation of 0.79. In addition, we found that for the reliability and consistency in the respondents’ responses regarding all statements dealing with employee abuse of managers – the Alpha-Cronbach index was high, which indicates high reliability. According to the survey data, we saw that there was no significant variation in the responses in gender distribution (t-index in exposure to workplace abusive behavior – 0.70), in the education of the respondents (variance test – exposure to workplace abusive behavior index – 0.90), and in the type of organization in which they work (variance test – workplace abusive behavior exposure index – 0.16).

Several findings emerged in the survey which we present below.

Associations between exposure to abuse and demographic indicators:

1. We found a relationship between the respondent’s age and seniority at work and some of the statements: A significant negative relationship (*r* = −0.203, *p* < 0.05) was found between the statement “Employee breaks promises s/he made to the manager” and age, so that the older the employee, the less they believe that employees break promises made to the manager. Similarly, it was found that the older the employee is (*r* = −0.288, *p* < 0.01) and the more seniority (*r* = −0.200, *p* < 0.05), the less likely s/he is to believe that the employee lies to his manager. In general, a significant negative relationship (*r* = −0.201, *p* < 0.05) was found between the index of exposure to abuse and age, so the older the employee, the less exposed s/he is to abuse.
2. Tenured employees agree more that an employee does not allow the manager’s interactions with his/her co-workers (1.97) more than an employee without tenure (1.58), and significantly: t (117) = 2.18, *p* < 0.05. No differences were found in the other statements and in the general index.
3. Managers are more clearly of the opinion than junior employees that employees invade the manager’s privacy, that employees blame the manager for their own mistakes, and that employees vent their anger on their managers for unrelated reasons.
4. Female managers are more likely than male managers to believe that employees lie to their managers. The survey data and the analysis of the findings show that there is indeed awareness of this phenomenon, although not in a clear way. This result of the exploratory study shows that many employees and managers are aware of the existence and various aspects of upward abuse as it is expressed in organizations, but they still do not give it an explicit name or are not willing to acknowledge it. The phenomenon of employee abuse of managers is not only not recognized in legislation (as is the phenomenon of regular workplace abusive behavior), but it is also not discussed in organizations and there are no procedures or processes to prevent and eradicate it.

## Data Availability

The raw data supporting the conclusions of this article will be made available by the authors, without undue reservation.

## References

[ref1] Bai Y. Lu L. Lin-Schilstra L. (2022). Auxiliaries to abusive supervisors: the spillover effects of peer mistreatment on employee performance. J. Bus. Ethics 178, 219–237. doi: 10.1007/s10551-021-04768-6

[ref2] Bhattacharjee A. Sarkar A. (2024). Abusive supervision: a systematic literature review. Manage. Rev Quarter. 74, 1–34. doi: 10.1007/s11301-022-00291-8

[ref3] Branch S. Ramsay S. G. Barker M. C. (2007). Managers in the firing line: contributing factors to workplace bullying by staff – an interview study. J. Manag. Organ. 13, 264–281. doi: 10.5172/jmo.2007.13.3.264

[ref4] Camps J. Stouten J. Euwema M. De Cremer D. (2020). Abusive supervision as a response to follower hostility: a moderated mediation model. J. Bus. Ethics 164, 495–514. doi: 10.1007/s10551-018-4058-0

[ref5] Cheyroux P. Morin A. J. S. Colombat P. Blechman Y. Gillet N. (2024). The effects of weekly levels of supervisor support and workload on next week levels of well-being, satisfaction, and performance as mediated by weekend work recovery. Stress Health 41:e3520. doi: 10.1002/smi.3520, PMID: 39676703 PMC11878750

[ref6] Choi Y. (2019). The moderating effect of leader member exchange on the relationship between workplace ostracism and psychological distress. Asia-Pacific J. Bus. Admin. 11, 146–158. doi: 10.1108/APJBA-11-2018-0205

[ref7] Eissa G. Lester S. W. (2017). Supervisor role overload and frustration as antecedents of abusive supervision: the moderating role of supervisor personality. J. Organ. Behav. 38, 307–326. doi: 10.1002/job.2123

[ref8] Escartín J. (2016). Insights into workplace bullying: psychosocial drivers and effective interventions. Psychol. Res. Behav. Manage. 9, 157–169. doi: 10.2147/PRBM.S91211, PMID: 27382343 PMC4924877

[ref9] Ferris D. L. Brown D. J. Berry J. W. Lian H. (2008). The development and validation of the workplace ostracism scale. J. Appl. Psychol. 93, 1348–1366. doi: 10.1037/a0012743, PMID: 19025252

[ref10] Fischer T. Tian A. Lee A. Hughes D. (2021). Abusive supervision: a systematic review and fundamental rethink. Leadersh. Q. 32:101540. doi: 10.1016/j.leaqua.2021.101540, PMID: 40260412

[ref11] Goldenberg-Aharoni S. Tziner A. Barnett D. (2019). Repercussions of incivility and hostile expressions in academia: a legal perspective. Ind. Organ. Psychol. 12, 385–390. doi: 10.1017/iop.2019.72

[ref12] Huh E. Lee E. S. Lee J. H. (2025). The roles of workplace ostracism and perceived power in predicting abusive supervision. J. Bus. Psychol. doi: 10.1007/s10869-024-10000-9

[ref13] Lev-Wiesel R. Barhon E. Itzkovich Y. Eliraz C. Shimony D. Goldenberg H.. (2023). Experiences of social workers who witness mistreatment as captured in drawing and narrative. J. Soc. Work. 23, 779–792. doi: 10.1177/14680173231164350

[ref14] Liang Y. Liu Y. Park Y. Wang L. (2022). Treat me better, but is it really better? Applying a resource perspective to understanding leader–member exchange (LMX), LMX differentiation, and work stress. J. Occup. Health Psychol. 27, 223–239. doi: 10.1037/ocp0000303, PMID: 34807679

[ref15] Lutgen-Sandvik P. Namie G. Namie R. (2010). Workplace bullying: causes, consequences, and corrections. Academia Edu. doi: 10.4324/9780203928554-9, PMID: 40143995

[ref16] Meiri E. (2013). The silent epidemic in workplaces – Bullying, harassment, maltreatment, psychological abuse: To know, to recognize, to cope. Hebrew: Effective Management Publications.

[ref17] Park J. (2023). The role of organizational efforts in mitigating the adverse effects of workplace mistreatment on attitudinal responses. Sustain. For. 15:1800. doi: 10.3390/su15031800

[ref18] Schaufeli W. B. Taris T. W. van Rhenen W. (2008). Workaholism, burnout, and work engagement: three of a kind or three different kinds of employee well-being? Appl. Psychol. 57, 173–203. doi: 10.1111/j.1464-0597.2007.00285.x

[ref19] Schneebaum G. (2021). Conceptualizing workplace bullying as abuse of office. South Carolina Law Review. Available online at:https://ssrn.com/abstract=3825692

[ref20] Tepper B. J. (2000). Consequences of abusive supervision. Acad. Manag. J. 43, 178–190. doi: 10.2307/1556375

[ref21] Tziner A. Bar-Mor H. Geva L. Levi H. Shkoler O. (2022). Abusive workplace behavior: behavioral and legal insights. Amfiteatru Economic 25, 235–250. doi: 10.24818/EA/2023/62/235, PMID: 27490260

[ref22] Tziner A. Shwartz Asher D. Yacovi T. Hatuel E. (2024). Peer abusive behavior: an exploratory study. Amfiteatru Economic. doi: 10.24818/EA/2025/69/643

[ref208] Weber M. (1978). Economy and Society: An Outline of Interpretive Sociology. Translated and edited by Guenther Roth and Claus Wittich.

[ref23] Woodrow C. Guest D. E. (2017). Leadership and approaches to the management of workplace bullying. Eur. J. Work Organ. Psy. 26, 221–233. doi: 10.1080/1359432X.2016.1243529

